# Histological evaluation of cellular response to a multifilament electrospun suture for tendon repair

**DOI:** 10.1371/journal.pone.0234982

**Published:** 2020-06-26

**Authors:** Mustafa Rashid, Jayesh Dudhia, Stephanie G. Dakin, Sarah Snelling, Antonina Lach, Roberta De Godoy, Pierre-Alexis Mouthuy, Roger Smith, Mark Morrey, Andrew J. Carr

**Affiliations:** 1 Nuffield Department of Orthopaedics, Rheumatology, and Musculoskeletal Sciences (NDORMS), University of Oxford, Oxford, United Kingdom; 2 NIHR Biomedical Research Centre, Oxford, United Kingdom; 3 Department of Clinical Sciences and Services, Royal Veterinary College, University of London, North Mymms, United Kingdom; 4 Department of Orthopedic Surgery, Mayo Clinic, Rochester, Minnesota, United States of America; University of Pittsburgh, UNITED STATES

## Abstract

**Background:**

Rotator cuff tendon repair in humans is a commonly performed procedure aimed at restoring the tendon-bone interface. Despite significant innovation of surgical techniques and suture anchor implants, only 60% of repairs heal successfully. One strategy to enhance repair is the use of bioactive sutures that provide the native tendon with biophysical cues for healing. We investigated the tissue response to a multifilament electrospun polydioxanone (PDO) suture in a sheep tendon injury model characterised by a natural history of failure of healing.

**Methodology and results:**

Eight skeletally mature English Mule sheep underwent repair with electrospun sutures. Monofilament sutures were used as a control. Three months after surgery, all tendon repairs healed, without systemic features of inflammation, signs of tumour or infection at necropsy. A mild local inflammatory reaction was seen. On histology the electrospun sutures were densely infiltrated with predominantly tendon fibroblast-like cells. In comparison, no cellular infiltration was observed in the control suture. Neovascularisation was observed within the electrospun suture, whilst none was seen in the control. Foreign body giant cells were rarely seen with either sutures.

**Conclusion:**

This study demonstrates that a tissue response can be induced in tendon with a multifilament electrospun suture with no safety concerns.

## Introduction

The prevalence of rotator cuff tendon tears is common (30%) in individuals over 60 years old [1]. Many are asymptomatic however, some become painful, with resulting loss of function, and may require surgical repair [2]. Over 17,500 rotator cuff repairs were performed between March 2015 and March 2016 in the National Health Service (NHS) in the United Kingdom [3]. Traditionally, rotator cuff repairs were performed via an open approach, using sutures grasping the tendon edge, passed through bone tunnels in the greater tuberosity, and secured with knots [4]. With improved understanding of the biomechanical environment and tear patterns, surgeons now employ a number of newer implant options and repair constructs, to help drive the best possible anatomical reconstruction of the tendon-to-bone interface [5–9]. Despite these advances, healing of the tendon to the bony footprint occurs in only 60% of cuff repairs and is influenced by age and tear size [10]. Compared to non-healed repairs, healed rotator cuff tendon repairs provide superior clinical improvement in patient reported outcomes [11,12].

Most surgeons opt for a combination of suture anchors, preloaded with sutures that are passed through the tendon. These can either be tied down (knotted repair), or hold the tendon in place without the need for knots (knotless repair) [13]. Some surgeons prefer to use a “double row” technique whereby the medial part of the tendon is attached with suture anchors to the medial part of the footprint and the free tendon edge is attached to the lateral part of the footprint using a second, lateral, row of anchors [5]. This double row technique can be linked to the medial row or unlinked, knotted or knotless, or a combination [13]. This evolution in surgical techniques and suture anchor technology has been accompanied by innovation in sutures used in rotator cuff repair. Generally, sutures used in rotator cuff repair are non-absorbable, braided, and much stronger than the native tendon [14, 15]. Recent innovations include suture tape, which has a wider surface area to better compress the tendon to the bony footprint [16]. Despite the aforementioned advances in techniques and implants, many of which have superior mechanical properties in a laboratory setting [17–20], clinically significant improvements to patient reported outcomes, or superior radiological healing rates, have not been demonstrated [21–23].

The location of failure following cuff repair can be at the anchor-bone interface, the tendon-suture interface (Type 1 failure), or from a recurrent tear, medial to the original repair (Type 2 failure) [24, 25]. The commonest mode of failure is from ‘cheese wiring’ of the suture through the repaired tendon. Whilst this has been reduced in biomechanical studies by using braided sutures to increase the coefficient of friction [16, 19, 26], the native tendon remains the vulnerable link. Currently, sutures used in rotator cuff repair are inert structures of synthetic material. They confer no favourable advantages to the native tendon other than mechanical ones, helping transfer load from the muscle to the bone, and providing compression of the healing tendon to the bone. This highlights the requirement for the development of bioactive sutures that are supporting the biological repair process of tissues. In particular, we hypothesise that it is possible to design new sutures with appropriate biophysical cues that positively influence tendon healing.

To create these biophysical cues in the suture material for tendon repair, aligned submicron fibres produced by electrospinning have shown promises [27–30]. These have the particularity of physically mimicking the native extracellular matrix structure and, as a consequence, of encouraging cell attachment, proliferation and differentiation (hence the term biophysical cues). We previously presented a method to produce continuous submicron electrospun (ES) polydioxanone (PDO) filaments, which can then be assembled into multifilament yarns serving as sutures. We showed a favourable cellular response *in vitro* and, following implantation in a rodent infraspinatus repair model, we demonstrated fibroblast infiltration and a transient foreign body giant cell (FBGC) response [28].

The aims of the current study were to investigate if the PDO electrospun suture could safely induce a positive tissue response and enable tendon repair in a surgical non-healing large animal model.

## Materials and methods

### Electrospun (ES) suture manufacture and characterisation

The ES sutures were produced in a similar manner as previously published [28]. Each step is briefly described in the following sections.

### Preparation of the electrospinning solution

Polydioxanone (PDO, 1.5–2.2 dl/g, Evonik, Essen, Germany) was dissolved into 1,1,1,3,3,3-hexafluoroisopropanol (HFIP 99.9% purity, Halocarbon Products Corporation, Atlanta, GA, USA) at concentrations of 11% (weight to volume ratio). The solutions were agitated at room temperature on a roller for at least 24 hours prior to use to allow for complete dissolution of the polymer granules.

### Electrospinning filaments

Electrospinning was performed in an enclosed container using a single nozzle high voltage power supply system (30 kV, SL30P30/230, Spellman, West Sussex, UK) and a syringe pump (World Precision Instruments Limited, Florida, US). Filaments were produced by using a thin stainless steel wire (100 μm in diameter, Goodfellow, Huntingdon, UK) as a collector. The wire was moved underneath the nozzle at a speed of 0.5 mm/s with a bespoke winding unit. The distance between the nozzle and the wire was 20 cm, and the average voltage applied was 6.5–7.3 kV. Electrospun filaments were then continuously wound up onto a motorised filament spool, separating them from the wire collector.

### Filament drawing and multifilament assembly

The collected electrospun filaments were drawn manually to produce stretched filaments. These were then twisted into a plied yarn in the “S” direction, using 5 filaments per ply. Cord yarns were then produced by twisting 7 plied yarns together in the “Z” direction. A twist ratio of 2:1 (S:Z) turns per meter was maintained throughout all samples.

### Annealing and sterilisation

Cord yarns were annealed at 65 ^o^C for 3 hours and cut to size (approximately 30 cm) to obtain the ES suture. All samples were stored in a desiccator at room temperature until implantation. Prior to implantation, sutures were sterilised by 70% ethanol for 6 hours.

### Scanning electron microscopy (SEM)

Samples were analysed, after gold coating using a SC7620 Mini Sputter Coater System (Quorum Technologies Ltd, East Sussex, UK), by scanning electron microscopy (SEM) using a Zeiss Evo LS15 Variable Pressure Scanning Electron Microscope (Carl Zeiss Microscopy GmbH, Oberkochen, Germany). Images were taken in triplicates at 5000X magnification to allow a minimum of 30 fibres to be measured for their diameter.

### Ovine non-healing intra-synovial model: Surgical procedures and macroscopic observations

The study was performed under UK Home Office license (PPL 70/6105) and with approval from the Ethics and Welfare Committee of the Royal Veterinary College (RVC). In a validated model [31], 8 female, skeletally mature, English mule sheep underwent tenoscopic surgery of the digital sheath. Using a 2 mm hook knife (ECTRA II disposable triangle knife, Smith and Nephew, UK), introduced through a proximal portal, a longitudinal surface defect (5 mm long; 2 mm deep) was created in the deep digital flexor tendon (DDFT) within the digital sheath, palmar to the metacarpophalangeal (MCPJ) joint in the right forelimb. The surgical defect was created in the coronal plane of the lateral branch of the DDFT only, which has been shown to persist over a 6 month period [31]. All surgeries were performed under general anaesthesia induced with xylazine, ketamine, and midazolam. Anaesthesia was maintained with ~2% isoflurane gas.

**Fig 1:**
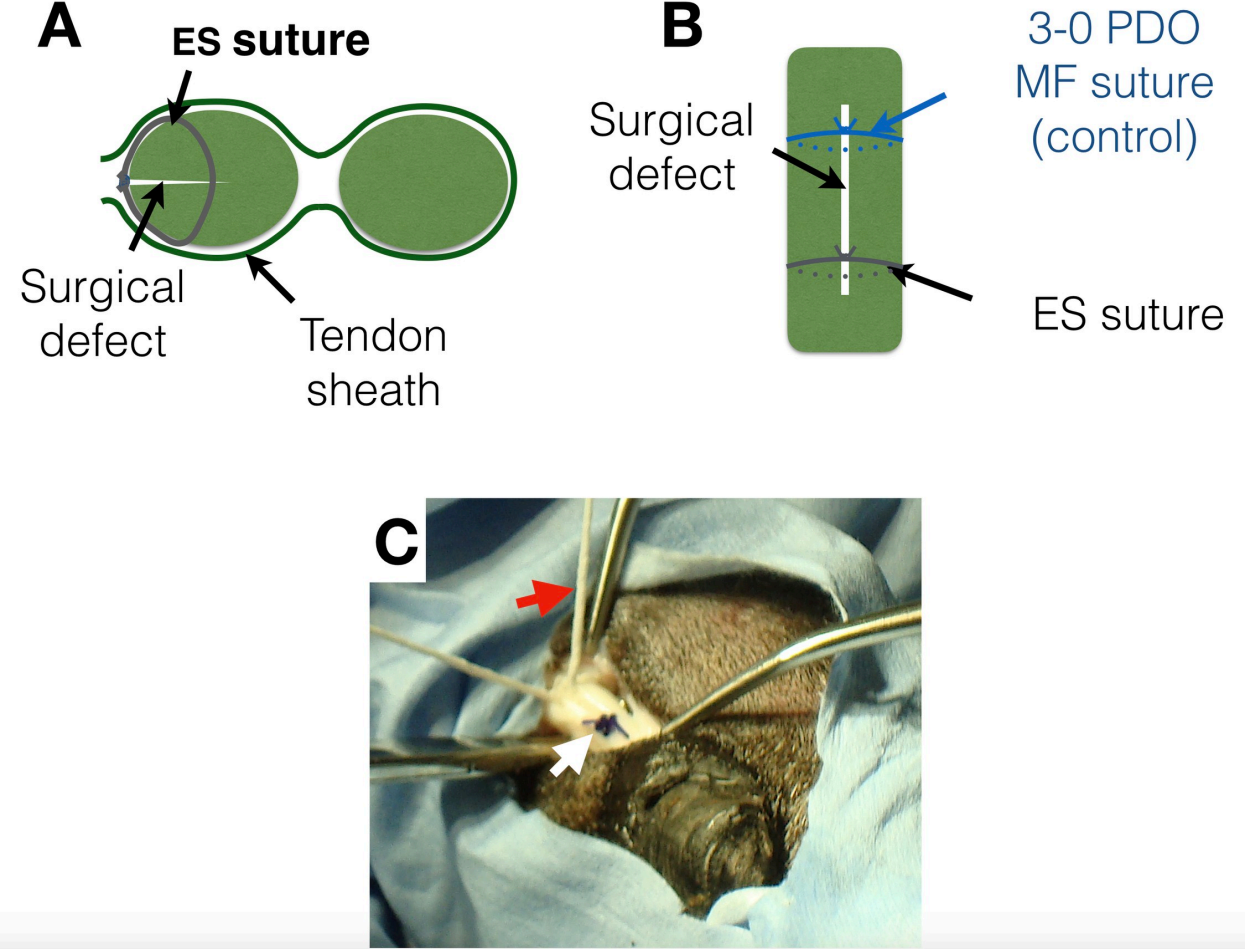
Electrospun (ES) suture and monofilament (MF) suture repair in an ovine model. A) Schematic diagram showing the transverse section of the deep digital flexor tendon (DDFT) repair model. B) Schematic diagram showing lateral view of the defect in the DDFT repaired with the MF suture (control) and the ES suture. C) Digital photograph showing surgical repair of the DDFT defect showing the ES suture (red arrow) and MF suture (white arrow).

Via enlargement of the proximal portal to expose the tendon defect, eight sheep had a repair with a 3–0 monofilament PDO control monofilament (MF) suture (PDS II, Ethicon, Johnson and Johnson Medical, Livingston, West Lothian, UK) and a PDO electrospun (ES) suture across the tendon injury using a simple surgeon’s knot, as shown in Fig 1. At the same time, the palmar annular ligament was transected to allow space for the suture knots to pass within the fetlock canal.

At 3 months, all sheep were euthanased by overdose of pentobarbitone at a dose of 1ml/kg body mass. Necropsy was performed in all sheep according to standard protocols. During tendon harvest, all repairs were examined macroscopically for signs of tissue healing, residual inflammation, knot retention, synovial fluid changes, and adhesion formation. The general appearance of the surrounding DDFT sheath and adjacent superficial digital flexor tendon was also recorded. Circumferential measurements proximal and distal to the MCPJ were made to assess swelling of the tendon repair site.

### Haematology and serum inflammatory markers

Blood samples for haematology testing were taken to rule out a systemic, infective or inflammatory response. Samples were collected from all sheep pre-surgery and 3 months post-surgery. Full blood count and serum inflammatory markers (serum amyloid A and fibrinogen) were performed by the clinical pathology laboratory at the Royal Veterinary College (RVC).

### Histology

The operated lateral branch of the harvested DDFT was transected at the tendon bifurcation and fixed in 10% formalin for 7 days. Tendon samples were then processed using a Leica ASP300S tissue processor and embedded in paraffin wax. Using a rotary RM-2135 microtome (Leica Microsystems Ltd, Milton Keynes, UK), 5–7 μm sections were cut and placed onto Surgipath X-tra Adhesive glass slides (Leica Microsystems Ltd, Milton Keynes, UK) and stained with haematoxylin and eosin (H&E). High magnification images (100X) were taken using a Zeiss AX10 inverted microscope with an AxioCam HRc camera and Axiovision software (Zeiss, Cambridge, UK) in bright field mode from 3 distinct areas or zones in the tissue sections, namely the ES, MF and NT (normal tendon) zones. ES zones refer to tissue areas found in close proximity to fragments of the ES suture. MF zones relate to tissue areas lying immediately adjacent to the MF suture material (often removed during sectioning). NT zones refer to sites distant to the suture implants (ES and MF) and served as an internal control group.

Taking 6 random images per field, per sample, we used histomorphometric features to quantify immune cell (IC) count, fibroblast cell (FC) count, and foreign body giant cell (FBGC) count. Vascularity was evaluated semi-objectively using the following grading system:

0 = no signs of vascularity;
1 = occasional red blood cells (RBCs) seen, or one small vessel;
2 = several groups of RBCs, or more than one small blood vessel;
3 = extensive blood vessels, one or more very large vessel(s), or many RBCs seen in the field of view.

### Statistical methods

Descriptive statistics included median values and 95% confidence intervals (CI). Comparisons between zones (ES, MF, or NT), using median values for immune cell count, fibroblast cell count, foreign body giant cell count, and vascularity were performed. A Mann Whitney U test was applied to determine the differences between fields (ES, MF, and NT) in the 4 categories (IC/ FC/FBGC counts and vascularity) using Prism v7 (GraphPad, USA). The level of statistical significance was set at < 0.05.

## Results

### Electrospun suture morphology

**Fig 2:**
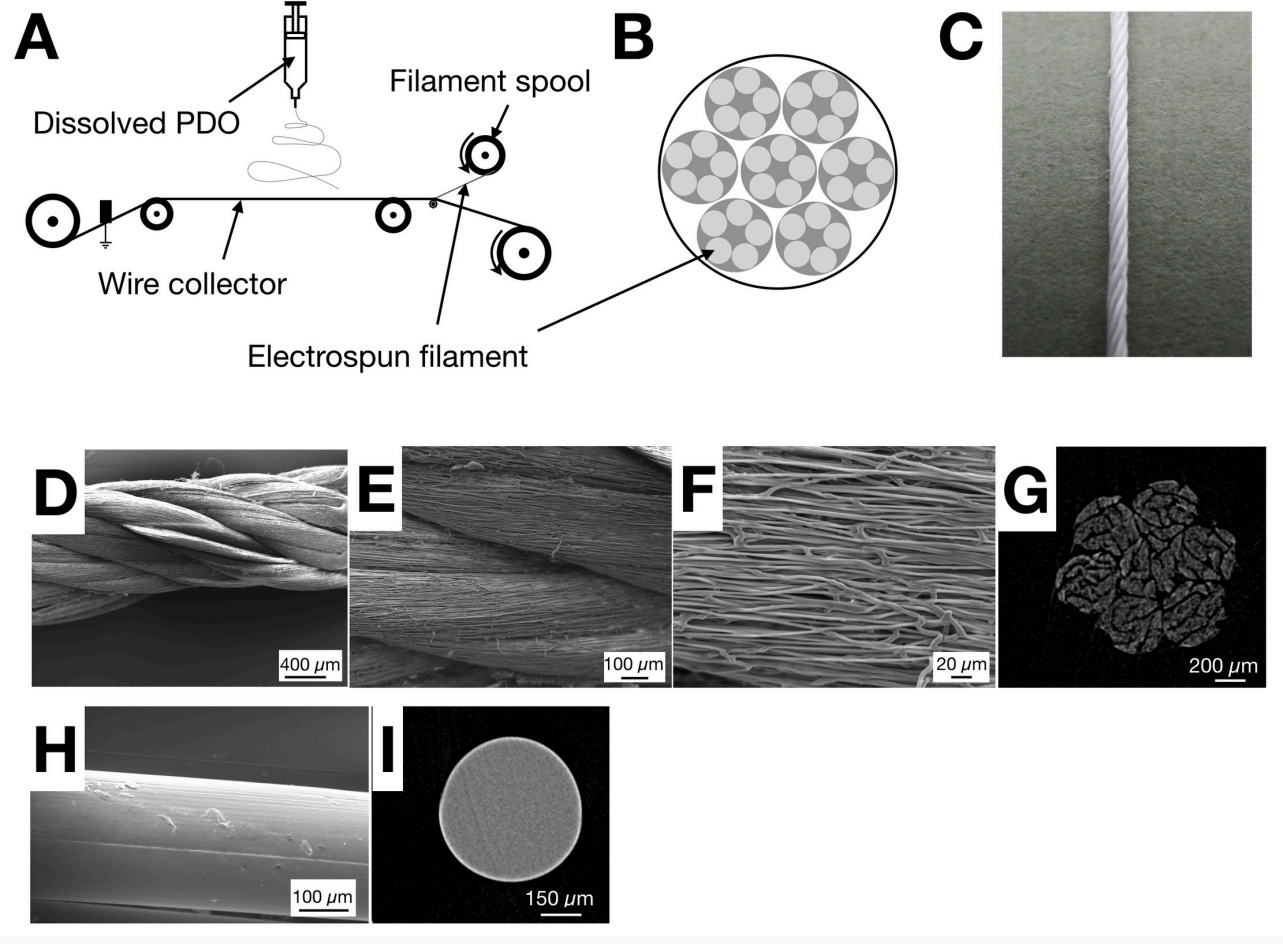
Production and characterisation of electrospun (ES) suture. A) Schematic illustrating the setup for producing ES filaments (building blocks). B) Schematic of the ES multifilament suture structure produced through the two-step twisting process. C) Digital photograph showing a complete ES suture. D-F) SEM images showing the twisted multifilament ES suture at different magnifications: 50X (D), 250X (E) and 1000X (F). G) MicroCT image showing the cross section of the ES suture. Note the similarity with the schematic shown in B and the spaces between filaments for potential cellular infiltration. H) SEM image of monofilament (MF) suture. I) MicroCT image showing cross section of monofilament suture (MF).

Multifilament electrospun (ES) sutures made of PDO were successfully produced with the method described above and sketched in Fig 2A and 2B. Characterisation using scanning electron microscopy (SEM) revealed the twisted multifilament structure (Fig 2D–2G), with electrospun microfibers aligned in the direction of the suture. The average diameter of the suture was 1.3 mm and the median diameter for the microfibers constituting the suture was 1.1 μm (IQR 0.8–1.3 μm). A micro computed tomography (microCT) image of the ES suture in cross section is shown in Fig 2G, indicating the individual filaments and the plied yarns separated by spaces of around 20 μm in width (later referred to as inter-filament space). In comparison, the 3–0 monofilament (MF) suture, was a dense, non-porous material as shown by the SEM and microCT images in Fig 2H and 2I, respectively. The average diameter of the MF suture was 0.3 mm.

### Macroscopic observations

**Fig 3:**
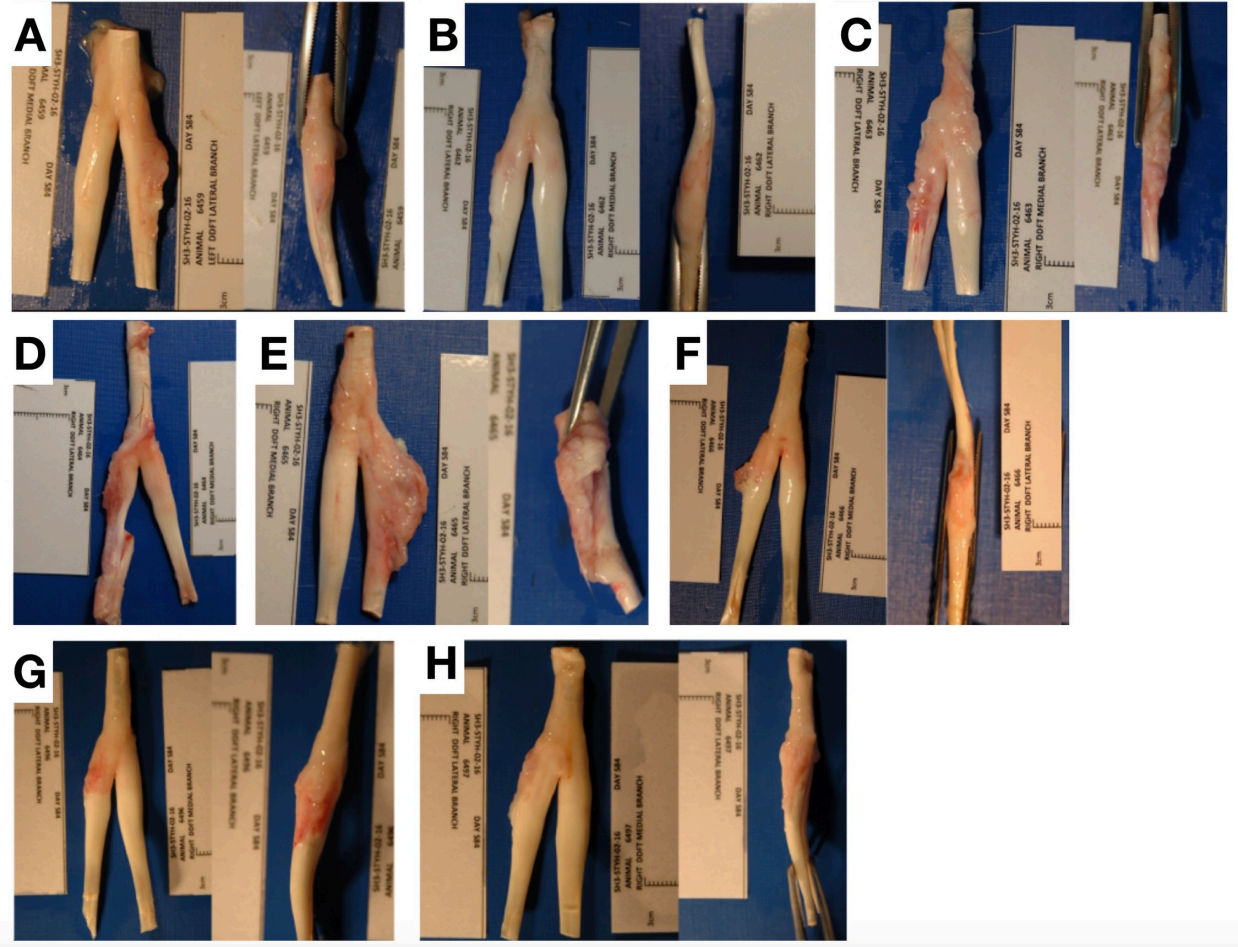
Macroscopic appearance of ES suture implanted ovine tendons. Digital photograph images showing harvested tendons augmented with the ES sutures at 3 months post repair. Sheep 6459, 6462, 6463, 6464, 6465, 6466, 6496, and 6597 are shown in A-H respectively. Proximal is to the top of the image.

None of the sheep showed signs of infection or tumour at necropsy. There was no difference in the circumference of the forelimb around the repair site (above and below the ergot) measured pre-surgery and 3 months post-surgery (see S1 Table). Macroscopic inspection of the lateral branch of the deep digital flexor tendon demonstrated an absence of any surgically induced tendon defect in all sheep, 3 months after the ES and MF suture implantation (Fig 3). There was no visible residual polymer from either suture in any specimens, although two sheep formed a fibrous capsule around a retained surgical knot from the ES suture (Fig 3D and 3E). The contour of the healed tendon over the created defect was altered. A smooth yet irregular lateral border with hyperaemia was observed in all specimens. All sheep demonstrated a normal amount (<0.5 ml) of synovial fluid with normal appearance, mild or no adhesion to the sheath and a local inflammatory reaction.

### Haematology and serum inflammatory markers

Haematology testing of all sheep for full blood count (including white cell count, neutrophil count, lymphocyte count, and red blood cell count) demonstrated no significant change. Serum markers for inflammation (serum amyloid A and fibrinogen) did not vary at 3 months post-surgery compared to pre-surgery levels (see S2 Table).

### Tendon histology

**Fig 4:**
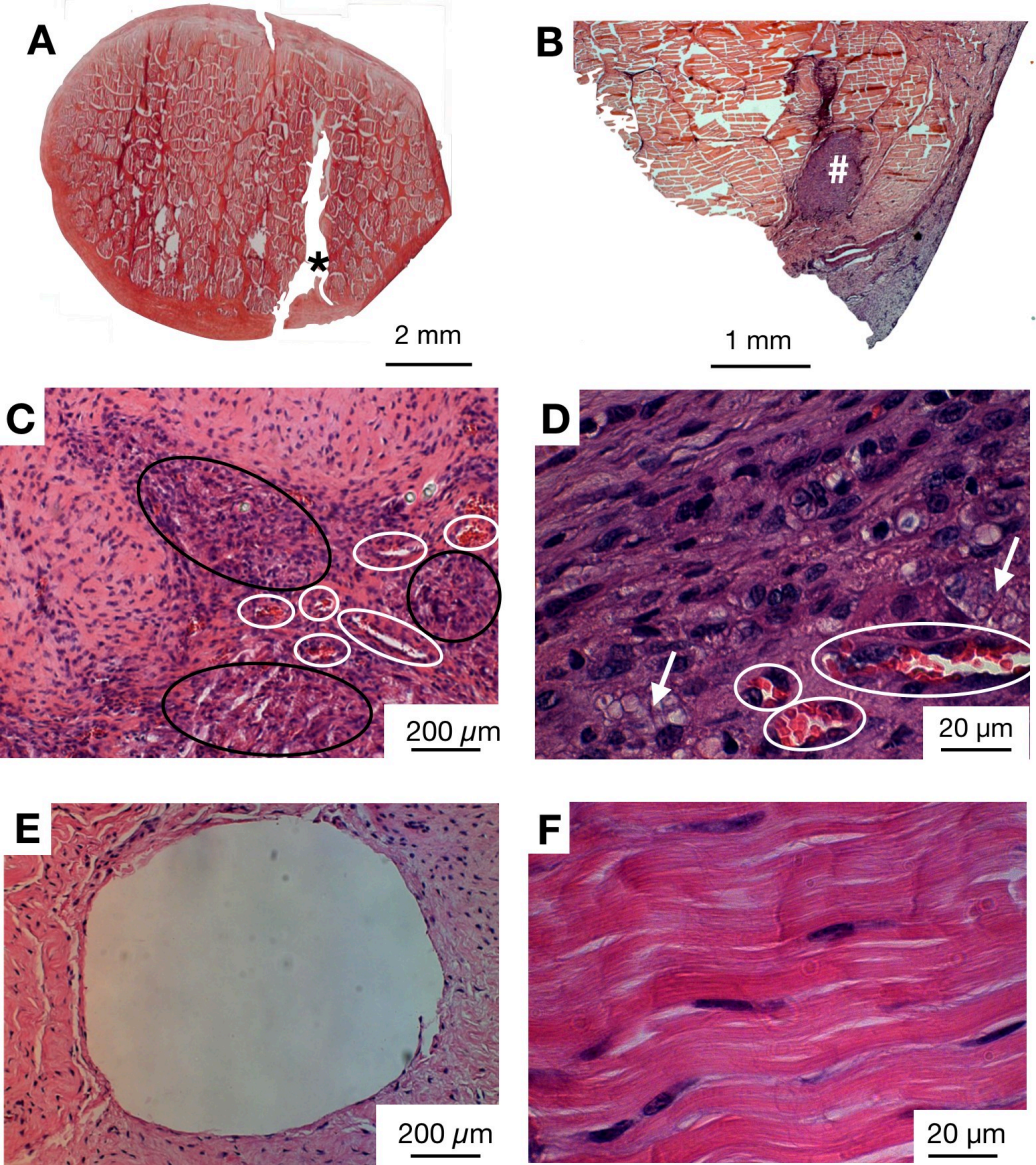
Histological appearance of ovine ES suture implanted tendons. A) Representative histology image (H&E stain, adapted from [31]) of cross section of DDFT tendon injury, 3 months after surgically-induced injury, showing persistence of the lesion (*). B) Histology (H&E stain) image of cross section of DDFT, absence of defect and presence of the ES suture (#). C-D) Histology image (H&E stain) showing zones filled with fibroblast-like cells (black ovals) within the area of the implanted ES suture, as well as blood vessels (white ovals) at magnifications (C) 10X and (D) 100X magnification. The white arrows indicate polymer fragments resulting from the ES suture degradation. E) Histology image (H&E stain) showing a complete circular defect in tendon left by the control suture (extracted by microtome), lacking cellularity and vascularity. F) Histology image (H&E stain) at high magnification (100X) of a normal tendon section showing fibroblasts sitting in a characteristic crimped extracellular matrix.

**Fig 5:**
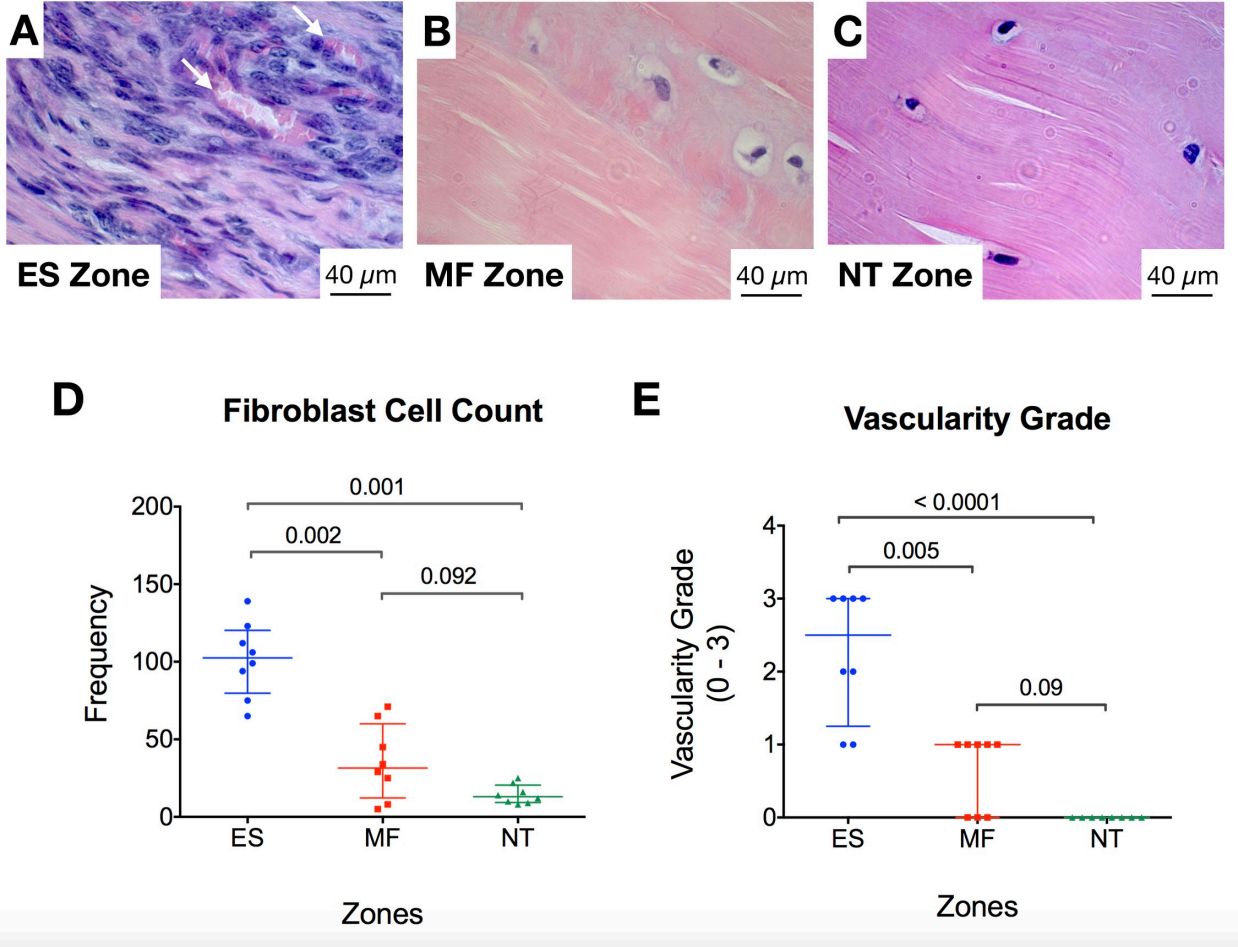
Cellularity and vascularity of ES suture implanted tendons. A-C) Bright field microscopy images showing the ES (electrospun) zone in A, the MF (monofilament control) zone in B, and the NT (normal tendon) zone in C. Note the high concentration of proliferative fibroblasts and the blood vessels (white arrows) seen in A. Also note similar appearance of scant well-orientated fibroblasts and no blood vessels in MF and NT zones. D) Graph showing median fibroblast cell count in ES, MF, and NT zones. E) Graph showing median vascularity grade in ES, MF, and NT zones. P values (Mann Whitney U test) are indicated to show the level of significance between zones.

Haematoxylin and eosin (H&E) stained sections demonstrated several key features, as shown in Fig 4. The ES sutures represented an area of highly cellular and vascularised tissue (Fig 4B–4D). The MF control sutures were visible in the sections of 3 of 8 specimens, and represented a defect devoid of any cells and without any increase in vascularity. The other 5 specimens demonstrated areas where the MF suture was extracted from the sample during sectioning with the microtome (Fig 4E). Some mild change to the tissue immediately adjacent to the MF sutures was observed, specifically an increase in fibroblast cell count. This zone was surrounded by an area of tissue with a normal-like tendon appearance, characterised by few, well-aligned fibroblast cells within a crimped extracellular matrix (Fig 5A–5C).

In both the ES and MF suture zones, FBGCs were rarely seen. A network of blood vessels supported the tissue integrated into the ES suture only. The neovascularisation was observed in the ES zone but not in the MF and NT zones.

As shown in Fig 5D and 5E, quantitative analysis showed that the ES zone was more vascular than the NT and MF zones (median vascularity grade ES 2.3 CI 1.5–3.0 vs MF 0.6 CI 0.2–1.1 vs NT 0 CI 0, p < 0.00001 and p = 0.09, respectively). The median number of fibroblasts in the ES zone (103, 95% CI 81–122) was significantly greater than in the MF zone (32, 95% CI 15–55) and NT zone (13, 95% CI 9–20, p = 0.002 and 0.001 respectively). There was no difference in the median FBGC count and IC count in all three zones (Median 0, FBGCs seen in ES, MF, and NT zones).

### Adverse events

**Fig 6:**
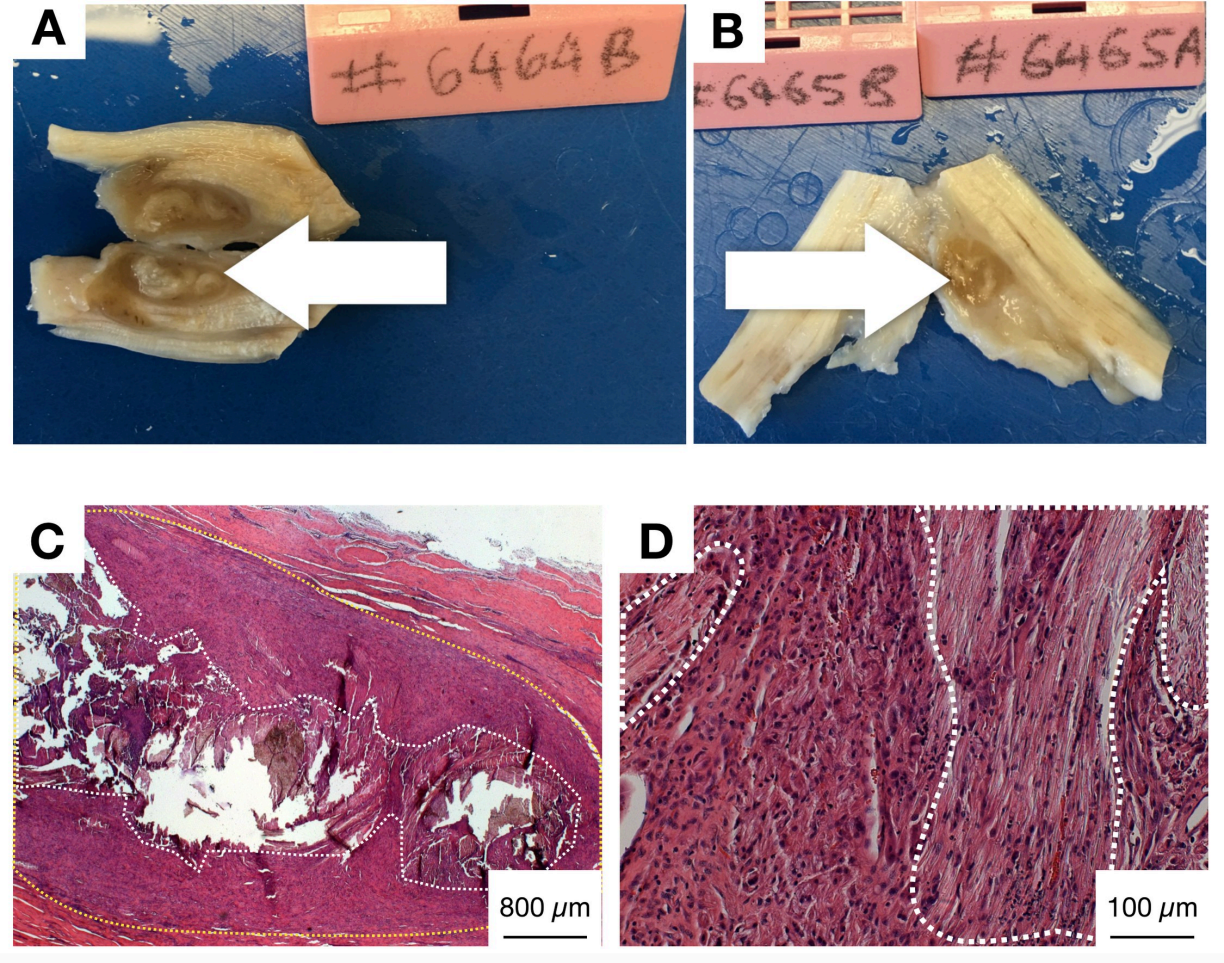
Explanted tendons with retained ES suture knots. A, B) Digital photographs showing retained ES suture knot (white arrows) adjacent to DDFT tendon, encapsulated by fibrous capsule. These were observed in two sheep, which both were active and did not demonstrate any lameness postoperatively. C) Low magnification (2.5X) bright field microscopy image of H&E stained tendon harvest sample from sheep 6464 showing retained degrading polymer (dotted white boundary), surrounded by area of highly cellular tissue (dotted yellow boundary). D) Higher magnification (20X) bright field microscopy image of the same sample as in C, showing the degrading ES suture material (dotted white boundary). Fibroblast-like cells seen in these areas were more aligned than in regions where the material was not visible (outside dotted white boundary).

Two sheep demonstrated post-operative lameness and were euthanased at 1 month post-surgery in accordance with standard protocols. This was due to irritation from the distal suture knot (the control monofilament suture in both sheep) within the relative constricted area of the tendon sheath at the level of the metacarpophalangeal joint fetlock canal. The MF suture was subsequently inserted proximal to the control suture in further sheep to avoid similar problems. All other sheep showed normal activity and behaviour and recovered uneventfully from surgery. Two other sheep, who were active and mobile following surgery, were noted to have a retained knot within a fibrous capsule (Fig 6A and 6B). On histology, these retained knot areas, adjacent to the healing tendon, were infiltrated with fibroblast cells (Fig 6C and 6D).

## Discussion

The ES suture demonstrated both safety and efficacy in an ovine model of surgically induced tendon injury. All tendon repairs healed on macroscopic inspection, with a mild local inflammatory reaction, minimal adhesions, and no excessive synovial fluid. No systemic response was seen on haematology, serology testing, or at necropsy. Compared to the MF control, the ES suture demonstrated significantly greater cellular infiltration and neovascularisation. In total four adverse events were encountered. Two events were due to the distal position of a large MF control suture knot causing local irritation within the constricted region of the tendon sheath, even after transection of the palmar annular ligament. Two further sheep demonstrated a retained ES suture knot within a fibrous capsule around a healed repair, these sheep were active and mobile without lameness.

Three months after surgical repair, the ES and MF sutures showed markedly different cellular responses on histological examination. In contrast with the MF sutures, which did not integrate into the host tissue, ES sutures were completely infiltrated with predominantly proliferative fibroblasts, and scant immune cells. It is worth noting that confirming the fibroblast phenotype remains challenging as distinct markers of ovine tendon fibroblasts are lacking in the literature. However, many of the cells populating the ES suture implanted tendons are large and exhibit fibroblast-like morphology. These are quite distinct from neutrophils, monocytes or lymphocytes which can be readily identified on H&E stained sections.

The difference in cellular infiltration between the 2 sutures was expected given the high porosity of the ES material, estimated to be at around 70% based on previous work [32], compared to the non-porous MF control (produced by melt extrusion). The role of the suture configuration, *i*.*e* multifilament (ES suture) versus monofilament (MF suture), has an obvious and important effect on tissue infiltration since the use of multiple filaments introduce porosity in the structure. While tissue is known to easily infiltrate in commercially available multifilament sutures [33], multifilament yarns that are too dense can prevent tissue infiltration: we have observed in a short pilot study that highly twisted ES sutures resulted in poor tissue infiltration compared to the looser ES sutures used in this study [34]. Our ES suture show an inter-filament space of around 20 μm in width which is suitable for cell infiltration as it is generally acknowledge that biomaterials with pores bigger than 10 μm enable cell infiltration [35]. Based on our previous work and the current study, we hypothesise that tissue infiltration occurs in 2 phases in the ES sutures: 1) During the first month following implantation, cells infiltrate the inter-filament space (*i*.*e*. between individual filaments within and around the plied yarns) due to the presence of large pores or conduits; 2) Following significant degradation of the PDO polymer in the later months, further cell infiltration occurs to occupy the space freed by the material. This is estimated to happen progressively between 1 to 5 months for the ES suture, with the suture fully degraded by 5 months [28]. For the MF control suture, tissue infiltration can only happen beyond 3 months, when the materials start to degrade significantly [36]. In this study, we observed an advanced state of degradation of the ES sutures at 3 months as polymer fragments could be seen in the tissue sections, while most of the MF suture material was still present (although if often detached during sectioning, leaving distinct gaps in the tissue). A faster degradation of the ES suture was expected compared to extruded MF sutures as they present a much larger surface to volume ratio, exposing the materials to more body fluid and therefore accelerating the hydrolytic degradation process [28, 37]. Other groups have shown similar cellular infiltration with a twisted electrospun polycarpolactone yarn to repair flexor digitorum longus tendon defect in a murine model [27], and an electrospun poly lactic-co-glycolic acid (PLGA) scaffold in an infraspinatus repair model in rats [38].

In the current study, we observed very few foreign body giant cells (FGBCs) around both ES and MF sutures at 3 months. Given the single time point, we do not know if FBGCs were present prior to the 3 month time point. However, in our previous study looking at the implantation of ES sutures in Lewis rats, we showed that FGBCs appear by week 6, and resolve to negligible levels by week 12 [28]. Using a different model, Kalfa and co-authors also showed a peak of FGBCs at 1 month in response to an electrospun PDO patch, that resolves completely by 3 months [39].

Accompanying cellular infiltration, the ES sutures supported a significant number of new blood vessels in the new tissue formed at the site of implantation. Tissue adjacent to the MF control did not demonstrate a significant rise in blood vessels compared to the normal tendon zones. Therefore, the increased vascularity in ES zones was determined to be due to inherent bioactivity rather than a reaction to the injury induced by implantation of a foreign material. Other studies have commented on neovascularisation in tendon repair after implantation of electrospun polymer tubes [40, 41], or electrospun collagen scaffolds [42]. One study made a comment of “vascular formation in the mid substance” of an implanted electrospun PLGA scaffold in tendon repair, but this was not quantified [38]. By contrast several studies have not shown any neovascularisation within electrospun scaffolds used in tendon repair, even when additional growth factors are loaded onto the scaffolds [27, 43]. Several other studies have applied electrospun scaffolds in other tissue types, including as vascular grafts, and demonstrated neovascularisation [44–46]. Other research groups have successfully stimulated neovascularisation by the addition of cytokines, growth factors, and stem cells to electrospun scaffolds [40, 47, 48].

At 3 months post repair, we noted a widening of the tendon contour reflecting a fibrous capsule surrounding a retained ES knot in 2 sheep. The ES sutures used in this study were of large diameter (1.3 mm), as these are intended for tendon repair in humans. Our model involved repair of a longitudinal defect in the deep digital flexor tendon, which is confined in a sheath and surrounded by synovial fluid. Whilst this made for an ideal model to investigate tendon repair as it is analogous to human disease where defects fail to heal without adequate intervention, it also meant that the ES suture, in its current form, may be too wide for this particular application in sheep. Histology of these two specimens showed that even the retained knot was heavily infiltrated with fibroblast cells and some foreign body giant cells.

The strength of this study is that we used a non-healing model of tendon injury in a large animal to assess efficacy [31]. No other small or large animal models of tendon injury has been developed to create an injury that consistently fails to heal without intervention. We acknowledge that we prioritised this aspect over biomechanical considerations, limiting our ability to comment on the mechanical performance potential of the ES suture. Unfortunately, non-healing models of tendon injury that offer a more mechanically relevant model of injury, *e*.*g*. involving loading perpendicularly to the injury, are not yet available. Moreover, we have not performed mechanical testing of the healed lesion because the primary purpose of the study was to investigate tissue response with histology. According to the literature, polydioxanone MF sutures retain about 50% of their original tensile strength after 6 weeks in vivo, 13% after 8 weeks and are absorbed in about 6 months [49, 50]. A similar decrease in strength was observed for our ES sutures incubated in phosphate buffer saline solution in previous *in vitro* work [28]. *In vivo*, while the degradation of ES sutures is expected to be faster (as mentioned earlier), the loss in mechanical properties is however predicted to be progressively compensated by the new tissue formed at the site of implantation [51]. Such advantage is not anticipated in MF sutures, at least within the first 3 months, as these do not allow for cell infiltration in that period of time. Another limitation in this study was the single time point for tissue analysis, meaning that it was not possible to determine temporal changes of the repaired tendon. Our previous study conducted in rats, did show cellular infiltration of tendon fibroblasts from week 1 to 20 post repair, as well as a transient FBGC response that peaks at week 4 and resolves to negligible levels by week 12 [28] which is likely to explain the lack of FBGC seen histologically in this study. A further limitation is that we selected PDS II sutures (extruded monofilaments) as our control. Sutures typically used in tendon repair are extruded multifilament yarns made of non-absorbable materials such as polyethylene terephthalate (*e*.*g*. Ethibond) or ultra-high molecular weight polyethylene (*e*.*g*. FiberWire®). While PDS II sutures are not available in a multifilament configuration, they are made of the same polymer (polydioxanone) as our ES suture. Given the profound impact of the chemistry of materials on the local tissue response, we believe PDS II sutures were a better control for this work. As mentioned earlier, there was also a large difference in diameter between the 3–0 control suture (0.3 mm) and the ES sutures (1.3 mm). We selected 3–0 sutures because they have a comparable strength to the ES sutures: their breaking force is reported to be around 50N [29, 30], which matches our ES sutures (unpublished data). The larger resulting diameter for the ES suture is a necessary compromise to achieve a highly porous structure that enables cell infiltration, while permitting effective surgical use without breakage. Finally, we were not able to comment upon the phenotypes of immune cells populating the implanted ovine tendons in this study. We extensively tested a panel of immune cell markers routinely used to characterise equivalent human cells, and found that these antibodies do not cross react with ovine proteins. We were unable to source commercially available antibodies for immune cell markers that cross-reacted with ovine tissues.

## Conclusions

The electrospun suture induced a tissue response predominantly involving tendon fibroblast-like cells at 3 months. We observed tendon repair in the non-healing intra-synovial ovine model with no evidence of abnormal local or systemic response.

## Supporting information

S1 Data (XLSX)

S1 Table Results of circumference measurements (in cm) of the right forelimb of the sheep taken above and below the ergot pre-surgery and pre-necropsy, 3 months post-surgery. (DOCX)

S2 Table Results of full blood count and serum inflammatory markers taken pre-surgery and pre-necropsy, 3 months post-surgery. WBC: White Blood Cells, Neut: Neutrophils, Lymph: Lymphocytes, RBC: Red Blood Cells.
(DOCX)
