# Supplementary material for: Histological evaluation of cellular response to a multifilament electrospun suture for tendon repair
Source: PLoS One. 2020 Jun 26;15(6):e0234982. doi: 10.1371/journal.pone.0234982 (PMC7319602; doi:10.1371/journal.pone.0234982)
Supplement: S1 Table — (DOCX) [file pone.0234982.s002.docx]

**Supplementary Table 1**: Results of circumference measurements (in cm) of the right forelimb of the sheep taken above and below the ergot pre-surgery and pre-necropsy, 3 months post-surgery.

|  | **Pre-surgery** | | **3 months post-surgery** | | |
| --- | --- | --- | --- | --- | --- |
| Ref. | MCPJ circumference - Proximal (cm) | MCPJ circumference - Distal (cm) | MCPJ circumference - Proximal (cm) | MCPJ circumference - Distal (cm) |  |
| 6459 | 11.0 | 10.5 | 12.0 | 11.5 |  |
| 6462 | 12.0 | 11.0 | 12.0 | 10.5 |  |
| 6463 | 12.0 | 11.5 | 12.0 | 13.5 |  |
| 6464 | 12.0 | 11.0 | 12.0 | 13.0 |  |
| 6465 | 12.0 | 11.0 | 12.0 | 11.5 |  |
| 6466 | 10.0 | 10.5 | 12.0 | 12.0 |  |
| 6496 | 11.5 | 11.0 | 11.0 | 11.5 |  |
| 6497 | 10.5 | 10.5 | 12.0 | 11.0 |  |
