# Supplementary material for: Histological evaluation of cellular response to a multifilament electrospun suture for tendon repair
Source: PLoS One. 2020 Jun 26;15(6):e0234982. doi: 10.1371/journal.pone.0234982 (PMC7319602; doi:10.1371/journal.pone.0234982)
Supplement: S2 Table — WBC: White Blood Cells, Neut: Neutrophils, Lymph: Lymphocytes, RBC: Red Blood Cells. (DOCX) [file pone.0234982.s003.docx]

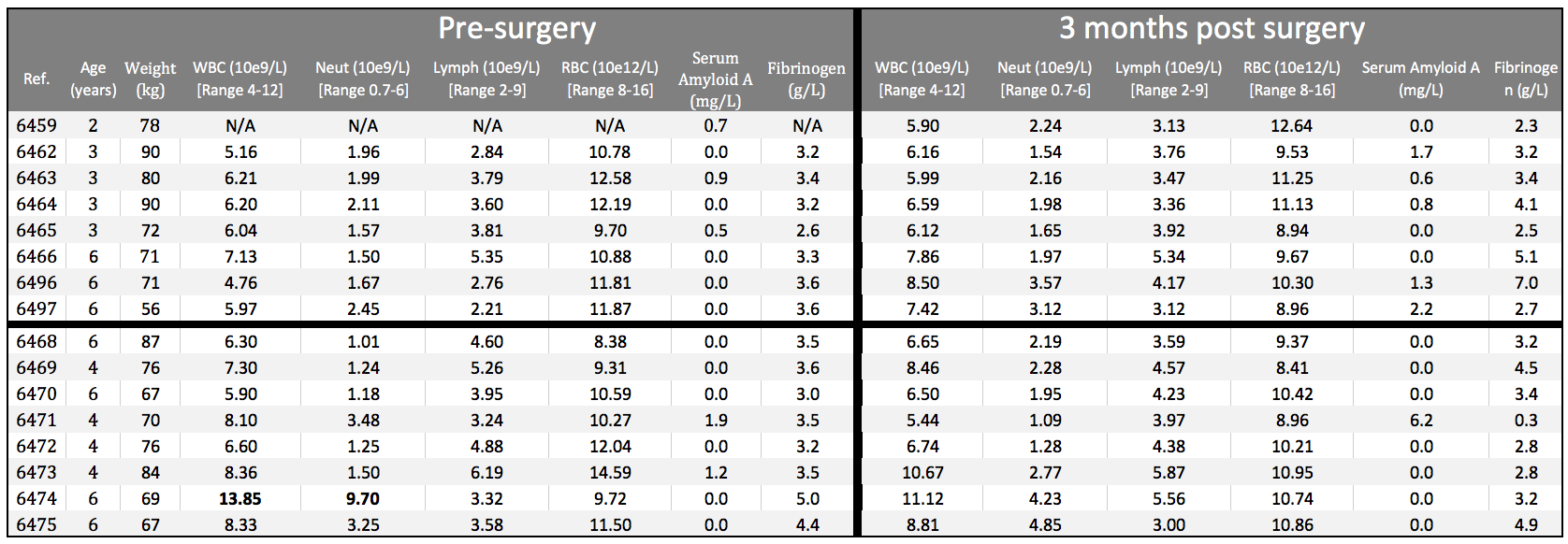
**Supplementary Table 2**: Results of full blood count and serum inflammatory markers taken pre-surgery and pre-necropsy, 3 months post-surgery. WBC: White Blood Cells, Neut: Neutrophils, Lymph: Lymphocytes, RBC: Red Blood Cells.
